# Grocery Shopping Under Simplified Marginal Value Theorem Predictions

**DOI:** 10.1007/s12110-024-09485-3

**Published:** 2025-01-17

**Authors:** Tabea Schlender, Alex Rieger, Frank Eggert

**Affiliations:** Institute of Psychology, Faculty of Life Sciences / Fakultät für Lebenswissenschaften, Technische Universität Braunschweig, Braunschweig, Germany

**Keywords:** Marginal value theorem, Optimal foraging, Human behavior, Supermarket, Behavioral ecology

## Abstract

This study examined whether supermarkets can be considered patches in the marginal value theorem (MVT) sense despite their particular features and whether they are models of human food foraging in resource-dense conditions. On the basis of the MVT, the quantitative relationship between gains in the Euro and patch residence time was modeled as an exponential growth function toward an upper asymptote, allowing the choice of an optimal strategy under diminishing returns. *N* = *61* participants were interviewed about their current shopping trip and contextual variables at a German supermarket and provided data to estimate relevant model parameters. A nonlinear model of the patch residence time and resulting gain based on an exponential function was fitted via nonlinear orthogonal distance regression. The results generally revealed the relationships predicted by the model, with some uncertainty regarding the estimation of the upper asymptote due to a lack of data from participants with long residence times. Despite this limitation, the data support the applicability of the MVT-based model. The results show that approaches from optimal foraging theory, such as the MVT, can be used successfully to model human shopping behavior even when participants’ verbal reports are used.

## Introduction

We investigate the application of the marginal value theorem to human food foraging behavior in urban societies. The main goal is to extend the area of application of this model to shopping behavior in resource-dense patches. A secondary goal is to provide an account of theory-based model building in terms of established biological theory using the example of shopping in a supermarket as foraging.

To accomplish these goals, we first review the conceptual and formal basis of the MVT as a representative model of optimal foraging theory. We survey the literature on successful applications of the MVT and then discuss specifics of human foraging behavior. We examine discrepancies due to focusing on urbanized societies and particularly supermarkets. After illustrating the derivation of a simplified MVT model, we interpret it for the application considered here and formulate specific model-based hypotheses. We then test the hypotheses and interpret the results in the context of a general theory of behavioral selection.

## Theory

### Optimal Foraging and the Marginal Value Theorem (MVT)

Optimal foraging theory suggests that individuals employ strategies that maximize their evolutionary fitness when foraging for resources (e.g., Saad & Gill, 2000; Wells, 2012). A resource may be anything relevant to the fitness of the foraging individual (Parker & Stuart, 1976), such as mating opportunities (Parker et al., 1999), body fat reserves (Hurly, 1992) or even social information (Turrin et al., 2017). The corresponding currencies that are used to maximize evolutionary fitness range from fertilization rates, risk and starvation avoidance to the one most relevant to this study, the net rate of energy gain (Krebs & Davies, 1993; Stephens & Krebs, 1986). When searching for these resources, the optimal foraging behavior depends on the benefit of the resource gained, the costs of reaching the patch (which is the source of resources) and the characteristics of the patch, such as its (limit of) resource yield or density or the costs of procuring the resource. To forage optimally, a forager faces three main decisions: (a) prey choice (see Morin, 2012), (b) patch choice (e.g. Patenaude-Monette et al., 2014), and (c) how long to forage in a patch before moving on to the next one (Charnov, 1976; Schoener, 1971; Stephens & Krebs, 1986). The MVT is a model of patch choice but can also be applied to the characteristics of foraging behavior in one patch.

More precisely, when resources are distributed heterogeneously and in discrete patches, the MVT predicts that the optimal time to spend in a patch before moving on to another patch is the exact time—the marginal value—at which the gain of resources per unit time equals the cost per unit time. This is true just before the costs begin to exceed the gain per unit time. The gross benefit of the resource item, the cost and time of traveling between patches, and the cost and time spent foraging within a patch influence the net gain rate.

The main source of food, and thus of foraging, for most humans in Germany is supermarkets. The greatest difference from previously studied MVT situations is the artificially constructed, very high-density nature of this foraging environment. To our knowledge, the MVT has not yet been tested in high-density foraging situations such as grocery shopping. Demonstrating its applicability would be a considerable extension of the MVT and thus would apply not only to hunter-gatherer-style human foraging but also to artificial, high-density resource patches in urbanized societies. This extension is not only in line with other evolutionary approaches to consumer behavior (Saad, 2013, 2017) but also provides testable quantitative predictions of functional forms, which remain underexamined in the behavioral sciences (Borgstede & Eggert, 2023; Muthukrishna & Henrich, 2019).

To consider whether such extended applications are plausible, the assumption of the MVT should be reviewed in light of the correspondingly extended foraging context.

### The Formal Model of the Marginal Value Theorem and its Assumptions

The MVT is based on the following assumptions:

1. Resources are found in a heterogeneous distribution in the form of discrete *patches* (Charnov, 1976). In this context, Bettinger and Grote (2016) defined patches as areas in which an organism forages that are separated from other patches by areas that are devoid of resources. The forager does not forage in these areas and must travel through them.
2. The individual possesses knowledge about the profitability of a patch, such as the initial resource yield rate in the patch (Kilpatrick et al., 2021) or the distribution of resources, which enables the organism to strive for an optimal foraging rate (Koops & Abrahams, 2003).
3. The resources in the patch are depletable (Stephens & Krebs, 1986).

Charnov (1976) formally describes the following model: A forager $\:j\:(j\:=\:1,\:2,\:\dots\:,\:n$, where $\:n$ is the total number of foragers) spends time $\:t$ traveling between patches. During this time, the forager does not obtain resources. $\:T$ is the time spent foraging in a patch. During this time, the net gain $\:g\left(T\right)$ is obtained. For a fixed $\:t$, the forager can maximize his or her marginal fitness gains (cf. Borgstede, 2024) by leaving the patch after the optimal patch residence time $\:{T}_{opt}$ having gained $\:g\left({T}_{opt}\right)$ resources, at which point the forager maximizes the net gain rate $\:{E}_{net}$. The net energy gain rate within a single patch of type $\:i\:(i\:=\:1,\:2,\:\dots\:,\:m$, where $\:m$ is the total number of patches) is further influenced by the proportion $\:{P}_{i}$ of patches of type i encountered by the forager. Charnov (1976) proposed the following equation for the net energy gain rate $\:{E}_{net}$:

$$\:{E}_{net,j}=\frac{\sum\:_{i=1}^{m}{P}_{i}\bullet\:{g}_{i}\left({T}_{j}\right)-t\bullet\:{E}_{t}}{t+\sum\:_{i=1}^{m}{P}_{i}\bullet\:{T}_{j}}$$

Charnov (1976) focused on contexts in which a forager changes between foraging in *multiple different patches*. The rule for whether to leave a patch and travel to a new patch is easy: the optimal residence time in a given patch is $\:{T}_{opt}$. Thus, at $\:{T}_{opt}$, the forager’s net gain rate of energy per time spent in the patch is at its maximum. Assuming optimal behavior, $\:{T}_{j}={T}_{opt}$. We refer to the times mostly as $\:{T}_{j}$ since this notation emphasizes the times as chosen by the foragers and then tested for optimality.

**Fig. 1:**
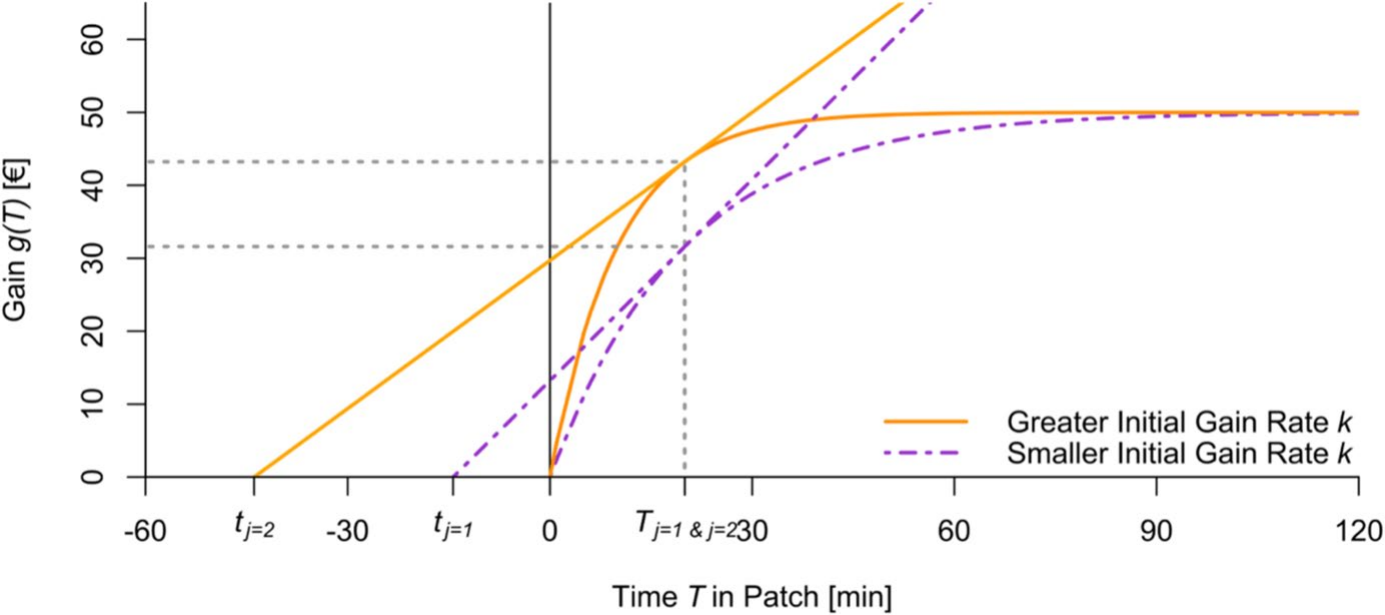
Different Patches, Each Modelled by Gain Curves and the Same Optimal Residence Time T. Note: Stylized curves for patches with different gain curves of the form $g_j\left(T\right)\;=\;A\cdot\left(1-e^{-k\cdot T}\right)$ . The functional relationship between different initial gain rates k and differences in travel time t_j_ for the same T_j_ and A.

For the same optimal residence time $\:{T}_{j}$ and different gain curves, the optimal travel time $\:{t}_{j}$ should be closer to 0 for a “poor” patch (with a smaller initial gain rate) than for a patch with high resource density or low costs in the patch (Fig. 1). For the same travel times to patches of different quality, higher costs must be evened out by higher gains in the patch, leading to higher optimal residence times.

**Fig. 2:**
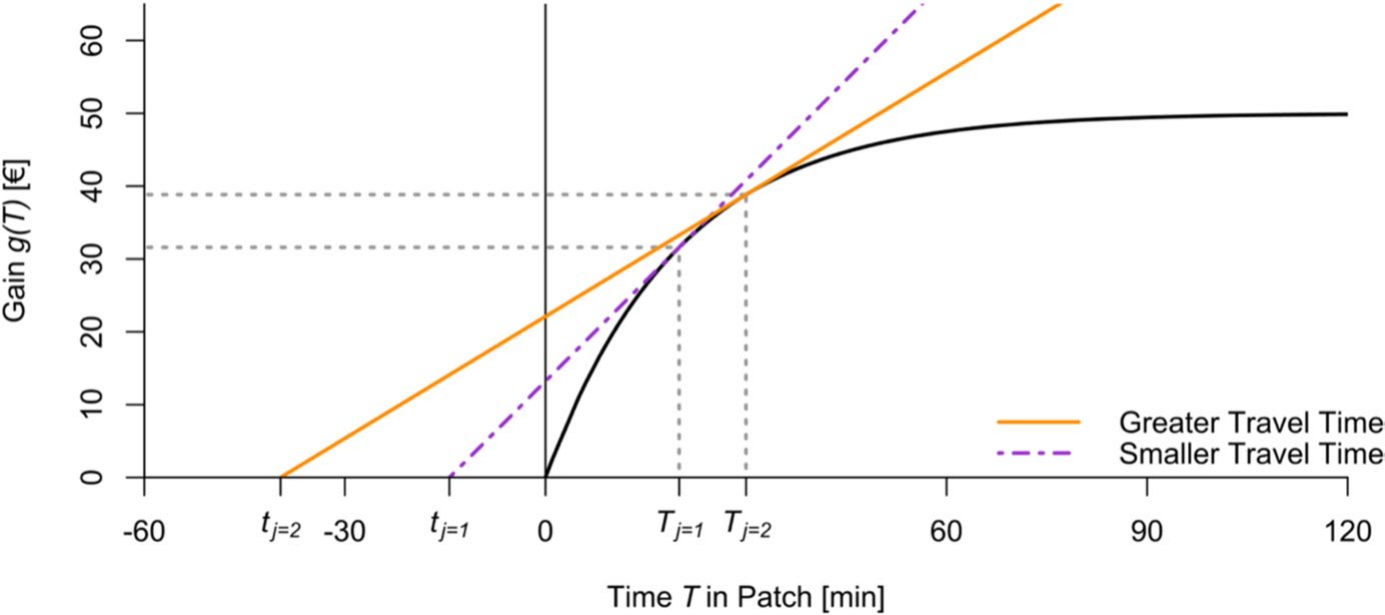
Different Travel Times t to the Same Patch Modelled by a Single Gain Curve. Note: The functional relationship between differences in travel time t_j_ and differences in the optimal patch residence time T_j_ for the gain curve of the form $g\left(T\right)\;=\;A\cdot\left(1-e^{-k\cdot T}\right)$ of a single patch.

The MVT applied to one patch (Fig. 2) predicts optimal patch residence times to maximize the net gain rate (Charnov, 1976). In a corresponding simplified model for individual foragers with one patch in which costs are operationalized solely via time expense, the energy gain rate $\:{E}_{net,j}$ in the patch can be found for each forager $\:j$ as

$$\:{E}_{net,j}\:=\:\frac{{g}_{j}\left({T}_{j}\right)}{{{T}_{j}\:-\:t}_{j}}$$

Note that $\:{t}_{j}\:$ is treated as a negative value in this study for better visualization of the model’s relationships (i.e., the origin is shifted for graphic purposes). Additionally, we now consider only one gain function g, modeling gain in a single patch.

A similarly simplified equation was used by Turrin et al. (2017), Wolfe et al. (2018) and Charnov and Parker (1995).

It is assumed that time $\:{T}_{j}$ that a participant $\:j$ spends in the patch is equal to the participant’s optimal residence time $\:{T}_{opt}$. Qualitatively, the model predicts that a longer travel time $\:{t}_{j}$ leads to the forager staying longer in the patch.

Stephens and Krebs (1986) described a curve of diminishing returns as the expectation for net gain when foraging and exponential growth toward an upper asymptote for gross gain.

### Empirical Support for the Marginal Value Theorem

Studies have shown that different foraging behaviors conform to the MVT predictions of patch residence times in patches and net gain rates, such as in mating (Parker, 1992) and food foraging (Cassini et al., 1990; Pyke, 1978; Todd & Kacelnik, 1993) With respect to mating behavior, Charnov and Parker (1995) argued that phenotypic variation affected travel time and gain rate. In their study, the copulation time of male dung flies was shorter and the proportional rate of sperm transfer was greater for larger males. Parker (1992) reported a good qualitative fit of the MVT in that the sperm reserves (gain) of dung flies increased with diminishing MVT returns during prey foraging. With respect to food foraging, Cowie (1977) demonstrated that the nonlinear functional relationship predicted by the MVT fits the average travel time and patch residence time for great tits (greater average patch residence times for greater average travel times) with artificial tree patches. Naef-Daenzer (2000) reported that the prey delivery rate of great tits depends on the size and density of their prey. In addition to these classical examples of MVT research in the past, the MVT has been tested on organisms in the classes of arthropods, birds and mammals. Hummingbirds abandoned a patch of flowers once the calculated departure threshold of a certain amount of nectar had been obtained (Pyke, 1978). Cassini et al. (1990) reported that the amount of patch exploitation for screaming hairy armadillos and guinea pigs varied with the patch quality or overall prey capture rate in accordance with the MVT but observed that MVT predictions remained below their observed patch exploitation. Wajnberg et al. (2000) also reported that the MVT can be applied to parasites: *Trichogramma brassicae* show longer patch residence times in higher-quality hosts and forage in all hosts to the same depletion level before leaving.

Furthermore, MVT relationships have been studied in the field. Hayward et al. (2015) collected data on the foraging behavior of wild European bison and reported higher greater abandonment of resources in patches with higher predation or poaching risks, such as areas close to roads or human habitation. Webber et al. (2021) found that caribou swimming between different islands left patches in line with predictions for MVT patch residence time. Assuming that island size is proportional to the amount of resources available, the MVT predictions can be confirmed: caribou visited smaller islands for shorter periods of time than larger islands. Even plant root growth seems to follow MVT foraging predictions: more plant root foraging can be observed in higher-quality soil patches, whereas no foraging efforts are observable when potential costs are higher than possible gains (McNickle & Cahill, 2009).

After reviewing the extensive empirical support for the MVT with nonhuman subjects, we would like to specifically draw attention to previous studies with human subjects, which is the focus of our application.

The MVT has been successfully applied to several foraging behaviors in humans, such as the reproductive strategies of men in rural Belize (Waynforth, 1999) and visual as well as internal search behavior (Wilke et al., 2009; Wolfe, 2013). On a more abstract level, the MVT explains that policies that regulate the availability of resources, such as regulating a child’s diet, promote overconsumption of these resources (Waynforth, 2010).

Studies that link human food foraging, MVT patch residence time and net energy intake rate predictions have focused primarily on hunter-gatherer groups foraging in nature. For example, Pacheco-Cobos et al. (2019) found a good fit of the MVT on the basis of the GPS tracks of mushroom foragers, which demonstrated continuous mapping between the encounter rate and search mode. Similarly, Venkataraman et al. (2017) showed a link between communal perceptions of resource depletion and decisions about movement in a hunter-gatherer society in accordance with the MVT. Some studies have also simulated foraging habitats virtually and analyzed human patch-foraging behavior in the context of the MVT (Constantino & Daw, 2015; Wolfe, 2013).

Notably, studies that have applied the MVT to human subjects to date have focused on human (optimal) foraging in natural environments. In contrast, a central element of the foraging behavior of humans in urbanized societies is the concentration of food resources in certain patches, such as supermarkets. Supermarkets have been regarded as patches in previous studies (Dickins & Schalz, 2020) and are, in some ways, similar to other structural situations to which the MVT has been applied: resources are concentrated heterogeneously in patches, and supermarkets are clearly distinguishable and separated from one another by areas devoid of groceries that must be traveled through. Additionally, individual knowledge about the profitability of the patch can be assumed because humans know the general spatial structure and general availability of most products due to similarities across supermarkets (Hwang et al., 2010). This can also be seen in customers’ frequent use of specific paths to selected aisles (Larson et al., 2005). Schwanen (2004) explored the determinants of shopping duration on workdays at a shopping destination (i.e., in a patch) without considering travel time, but no studies have focused on purchasing behavior in supermarkets in light of the MVT. However, some previous research has examined the MVT or optimal foraging in the context of online shopping behavior (Friske & Choi, 2013; Wells & Foxall, 2012).

### Supermarkets as Patches with Regard to the MVT

Studies such as the work by Cowie (1977) argue that the gain function should diminish over the course of the patch residence time because prey (or resources in general) are depletable. This is usually not the case for supermarkets, which have large amounts of resources and a certain amount of restocking. Thus, food as a resource is generally available in abundant amounts and, under normal conditions, is not depletable for the individual (e.g., ignoring panic buying during the COVID-19 pandemic (Dickins & Schalz, 2020). However, when we focus on resources and not just gains, resources such as money and, most importantly, carrying capacity are depletable and limited for customers. When a forager’s need for a resource is fulfilled, the benefit of the resource diminishes.

If the MVT does not depict the absolute amount of gain but rather the beneficial effects of the resource (which are always ultimately diminishing), even for unlimited resources, the result is a growth function toward an upper asymptote that makes the assumption of depletable patches irrelevant. This function occurs not only for depletable patches with limited resource availability but also for diminishing beneficial effects (i.e., predicted changes in fitness; cf. Borgstede, 2020; Borgstede and Eggert, 2021) of a resource. In particular, humans foraging in a supermarket usually have a list of items to be bought (either implicit or explicit) that usually include different units rather than several of the same item. For example, one might have a list of two portions of noodles, tomato sauce and mozzarella as well as bread, butter and gouda. Importantly, any useful set of substitutable (food) items leads to less importance of any subsequent items (i.e., with lower additional value). From a continuously idealized perspective, this implies a diminishing returns curve as in the MVT and, in general, a qualitatively similar increasing one that might have discrete jumps.

**Fig. 3:**
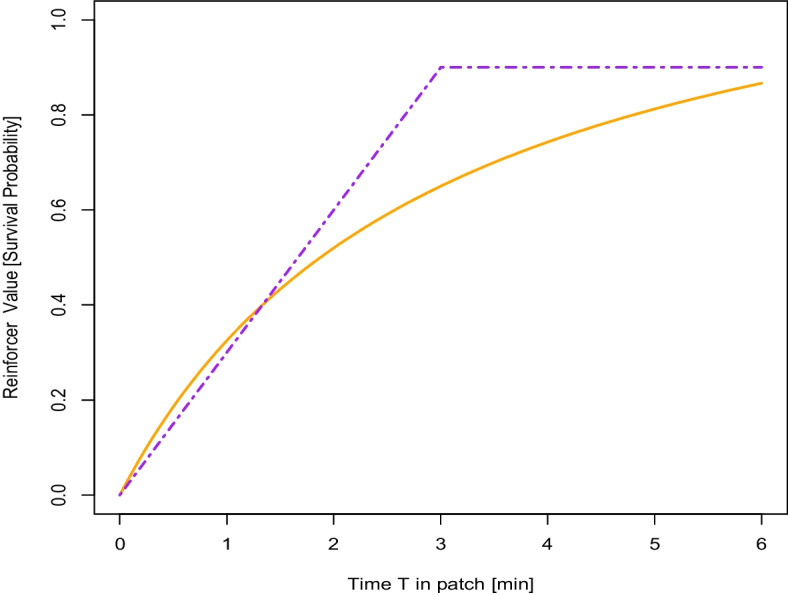
Possible Functional Relationships of Behavioral Allocation, i.e., Patch Residence Time and Resourced Gain in Terms of Marginal Fitness. Note: Two different possible relationships of marginal fitness consequences as a function of patch residence time are presented. The dotted line shows a curve resulting from a piecewise function where the gains are linear until there is a sudden drop. The solid line shows a smooth case of diminishing returns.

This can be explicitly modeled by a composition of two functions as in Borgstede (2020): a feedback function $\:F$ (Prelec & Herrnstein, 1978) to model the relationship of the behavior $\:{b}_{j}$ of individual j and its consequences and a function $\:P$ relating the consequences to marginal fitness (which could, for simplicity, be reduced to contributions to survival probability). Mathematically, this results in a model of the form $\:r\left({b}_{j}\right)=P\left(F\left({b}_{j}\right)\right)$. Depending on the characteristics of the patch and the forager, these functions can take several different forms, which are presented in Fig. 3. Interestingly, any plausible choice for the supermarket example necessarily leads to a sort of diminishing returns model. More precisely, a plausible assumption for the feedback function $\:F$ would be a function that is either linear with a sharp cutoff from which it is constant (signifying a plateau for an empty shelf; see the dotted curve in Fig. 3) or a diminishing returns curve by itself (if one forages for products that may be substitutable, e.g., from different brands or organic/nonorganic but with increasing effort required to find them; see the solid curve in Fig. 3). The function $\:P$, which models survival probabilities, would generally be assumed to have an upper asymptote (Borgstede, 2020), thus creating a marginal fitness curve with the same qualitative characteristics as those given by the MVT (Charnov, 1976). This perspective is analogous to that of Bird and Bird (2021), who described the effects of foraging in a patch on the rate of change in returns. Here, however, the approach leads to a different conclusion due to differences in the characteristics of the patch and the forager in the supermarket setting. While Bird and Bird (2021) described a case in which foraging in a patch increases returns, here, we detail how returns can be diminishing without the number of products in stock at the supermarket necessarily decreasing.

Nevertheless, there are some ways in which supermarkets may present a different foraging situation than traditionally assumed by the MVT.

The concept of patches is somewhat ill-defined in that most patches can be considered to be composed of different, smaller patches (see also the discussion in Pacheco-Cobos et al. (2019) on broadening the scope of MVT by moving away from the notion of necessarily discrete patches). Supermarkets can be considered to consist of interindividually different internal patch structures. For each forager, each product area can be considered a smaller patch within the supermarket patch. Following this idea, a forager would search in each smaller patch of the supermarket for different kinds of resources (e.g., toilet paper is a different unit than meat). However, at a more abstract level, all products in a supermarket are resources to be foraged, even if they are in different gain units. Therefore, another level on which supermarkets can be examined as patches is the focus on a single supermarket as one level of analysis.

One could note that most foraging situations are cryptic and stochastic in that resources must be searched for and acquisition is not certain, which is difficult to assume for supermarkets. While cryptic and stochastic foraging means reaching the upper asymptote only at presumably much longer patch residence times or with statistical noise, the model in the supermarket would still qualitatively retain a growth function toward an upper asymptote, as initially discussed by Charnov (1976). Winterhalder (1986) also demonstrated that optimality predictions in stochastic environments in which foragers minimize risks differ insubstantially from those of deterministic models in which foragers maximize their net gain rate. While completely deterministic shopping paths in the supermarket can occur, due to aspects of uncertainty, stochastic search processes are to be expected even in the clearly structured environment. Some products might be out of stock and require a search for alternatives, or a forager might forget some products and need to return to other parts of the supermarket.

Considering these aspects in which human shopping behavior differs from typical MVT and optimal foraging situations, the question arises whether supermarkets still fundamentally perform like MVT patches despite their characteristics. Is human food foraging in supermarkets in urbanized societies so different from classical foraging contexts that it can no longer be sufficiently described by the MVT?

## Modeling the Supermarket Setting by Applying the MVT

### Parameters and Measures for the Supermarket Setting

The general approach toward travel time, time in the patch and the gain or benefit of a resource is based on Charnov (1976). However, several assumptions are made for simplification that correspond to an existing reduced model by Charnov and Parker (1995). We discuss these simplifications in more detail since they illustrate the process of constructing a mathematical model of a complex and statistically noisy context.

Since it would be difficult to define different supermarkets as patches of the same type $\:i$, (e.g., due to varying assortments even between supermarkets of the same chain), we focused on a single patch; thus, $\:i\:=\:1$. This also implies that the encounter proportion for that single patch is set to $\:{P}_{1}=1$, effectively removing this parameter, as in Bella-Fernández et al. (2022) Eq. 4.

Frequently used measures of gain include biomass, calories of food per volume in kcal or ease of handling, as suggested by Schoener (1971). Since these are difficult to estimate for each type of product and participant, a simplified approach was chosen that uses the number of different products bought as well as the money spent as numeric measures of the benefit of a resource, making the gain rate of products the currency.

$\:{E}_{t}$ usually includes travel costs for traveling to the patch and back home (Charnov, 1976; Schoener, 1971). Travel costs depend on numerous other aspects, such as the weight of groceries, the physical constitution of the person or complex shopping in groups. For simplicity, we disregard these aspects for the moment.

For $\:{E}_{patch}$, we make similar simplifying assumptions. While in other foraging contexts the costs within the patch may include capturing, handling, processing, and consuming the resource (Morin, 2012; Schoener, 1971), foraging in urbanized supermarkets usually does not include these aspects. Thus, we abstract away from more detailed analyses of, for example, caloric costs.

Due to the constraints, $\:{E}_{t}$ and $\:{E}_{patch}$ are not included in the simplified MVT model. Since we simplify assumptions of individual foragers’ characteristics such as physical carrying capacity, we assume only one common gain curve within the patch for groups of participants with shared characteristics, corresponding to Fig. 2.

As a result of the considerations above, the predictions of the simplified model depend only on travel time $\:{t}_{j}$ without other subject-specific predictions. We use time as a single indirect and commensurate measure of costs.

*Variable *$\:t$ is then the *time spent traveling* to the patch or supermarket in minutes. It consists of the sum of the time for travel to the supermarket and travel home (Krebs & Davies, 1993). Here, it is important to pay attention to subgroups that emerge when studying human foraging in urbanized environments. Most importantly, the use of transport vehicles (e.g., cars, busses) at least partly decouples the otherwise proportional relationship of carrying capacity and energetic costs while also introducing more complex trade-offs (for example, prices for fuel or bus tickets). However, for transport by foot, humans are limited by the maximum transport quantity (Jones & Madsen, 1989). In previous studies of the MVT, movement patterns were tracked using GPS points at fixed time intervals and the linear distance (Naef-Daenzer, 2000; Pacheco-Cobos et al., 2019; Patenaude-Monette et al., 2014) was calculated, which was not possible here because of the study of foraging behavior in a natural setting. A previous study (Lange et al., 2014) reported that the subjective perception of distance traveled to recycling sites, which can be interpreted as patches, influenced recycling behavior more than the linear distance did. Therefore, we aimed to include distance in km estimated by participants as an operationalization of travel costs as well as Google Maps data for objective distance (Google Maps, n.d.).

*The variable *$\:T$ is the *time spent in the supermarket patch* in minutes. For predators, the time spent searching for and possibly pursuing prey within a patch is affected by acceleration and exhaustion (Schoener, 1971). Although shopping in a supermarket usually does not entail the effort of active pursuit, one may have to invest some effort into searching for particular items that are bought only at longer time intervals (e.g., spices). Thus, it seems sensible to inquire about whether participants perceive shopping as emotionally or physically exhausting because this may indicate increased costs of searching in the patch.

The resulting model by Charnov and Parker (1995) proposes an exponential decay of the distance to an upper asymptote for the gain curve:

$$\:g\left(T\right)\:=\:A\:\bullet\:\:(1\:-\:{e}^{-k\:\bullet\:\:T})$$

Here, $\:T$ is the time spent in the patch or patch residence time, $\:A$ equals the maximum amount of gain possible in the patch, and $\:k$ is the initial gain rate, corresponding to the slope of the linear approximation to the curve at the origin. The net gain rate $\:{E}_{n}$ at a given time $\:T$ is given by the first derivative $\:{g}^{{\prime\:}}\left(T\right)$ of the gain curve. Since the slope of a curve (its first derivative) equals the slope of its corresponding tangent line at any specific point, this can be employed to predict the corresponding travel time $\:{t}_{j}$ for any given optimal residence time $\:{T}_{j}$. This assumes a sufficiently smooth (i.e., differentiable) model function, as stated above. Technically, this is not true for the origin since the model function has a kink there. However, this is not considered relevant since it implies a patch time of *T = 0*, which would not lead to inclusion in the empirical study.

We now show how to calculate the travel time $\:{t}_{j}$:

The general equation for a tangent line is

$$\:{f}_{j}\left(T\right)\:=\:{g}^{{\prime\:}}\left({T}_{j}\right)\:\bullet\:\:\left(T\:-\:{T}_{j}\right)\:+\:g\left({T}_{j}\right)$$

The tangent line is zero at $\:{t}_{j}$; therefore,

$$\:{f}_{j}\left({t}_{j}\right)\:=\:0\:=\:{g}^{{\prime\:}}{(T}_{j})\:\bullet\:\:({t}_{j}\:-\:{T}_{j})\:+\:g\left({T}_{j}\right)\:$$

Solving for *t*_*j*_ yields

$$\:{t}_{j}\:=\:{T}_{j}\:-\:\frac{g\left({T}_{j}\right)}{g{\prime\:}\left({T}_{j}\right)}$$

This gives us a way of calculating $\:{t}_{j}$ if the other values are known or estimating it if the other values can be estimated.

However, instead of comparing observed and predicted travel times $\:t$, several studies compare predicted to observed optimal patch residence times $\:T$. An equation for this can be found as follows: since for all $\:j$, $\:g\left({t}_{j}\right)\:=\:0$, it follows that with a general y-intercept *b*,

$$\:0\:=\:g{\prime\:}\left({T}_{j}\right)\:\bullet\:\:{t}_{j}\:+\:b$$

$$\:\:b\:=\:-A\:\bullet\:\:k\:\bullet\:\:{e}^{-k\:\bullet\:\:{T}_{j}}\:\bullet\:\:{t}_{j}$$

The equation for the tangent line therefore is as follows:

$$\:{f}_{j}\left(T\right)\:=\:g{\prime\:}\left({T}_{j}\right)\:\bullet\:\:T\:-\:{g}^{{\prime\:}}\left({T}_{j}\right)\:\bullet\:\:{t}_{j}$$

$$\:{f}_{j}\left(T\right)\:=\:A\:\bullet\:\:k\:\bullet\:\:{e}^{-k\:\bullet\:\:{T}_{j}}\:\bullet\:\:T\:-A\:\bullet\:\:k\:\bullet\:\:{e}^{-k\:\bullet\:\:{T}_{j}}\:\bullet\:\:{t}_{j}$$

The optimal patch residence time and gain are given by the gain curve. Substituting $\:Q\:=\:({T}_{j},\:g\left({T}_{j}\right))$, the tangent equation yields $\:g\left({T}_{j}\right)\:=\:A\:\bullet\:[1\:-\:{e}^{-k\:\bullet\:\:{T}_{j}}]$

$$\:A\:\bullet\:\:\left(1\:-\:{e}^{-k\:\bullet\:\:{T}_{j}}\right)\:=\:A\:\bullet\:\:k\:\bullet\:\:{e}^{-k\:\bullet\:\:{T}_{j}}\:\bullet\:\:{T}_{j}\:-A\:\bullet\:\:k\:\bullet\:\:{e}^{-k\:\bullet\:\:{T}_{j}}\:\bullet\:\:{t}_{j}$$

$$\:{e}^{k\:\bullet\:\:{T}_{j}}\:=\:k\:\bullet\:\:\left({T}_{j}\:-\:{t}_{j}\right)\:+\:1$$

From this equation, the predicted optimal patch residence time $\:{T}_{j}$ can be found via iteration. This equals Eq. 4 in Charnov and Parker (1995); the value of $\:{t}_{j}$ is always negative in this study, whereas it is always positive in Charnov and Parker (1995).

Similarly, Putrevu and Ratchford (1997) assumed that shopping enjoyment lowers the costs of searching for items in a supermarket. Niemeier and Morita (1996) reported that humans shop for a shorter period when shopping on the way home from work compared to shopping activities that begin and end at home. Within the context of the MVT, this implies that for shopping while pursuing other activities, travel costs to the supermarket do not account for the entire way and the time traveled, leading to different optimal gains compared with travel from home to the supermarket and back. There are three main types of determinants of shopping duration:

1. Temporal constraints such as time pressure, work situation, or the number of household members one must care for can reduce shopping time in a supermarket (Niemeier & Morita, 1996; Putrevu & Ratchford, 1997; Schwanen, 2004; Wells, 2012). Additionally, shopping in a larger group of people may be more efficient for the participant (cf. Schoener, 1971) if the search for products is divided among all foraging group members, reducing the costs for the individual in the patch. A greater number of household members to provide for may increase the number of products that must be purchased.
2. Activities and travel episodes in the context of the shopping activity may determine the shopping duration, as described above (Schwanen, 2004).
3. Monetary budgets, such as higher household income, have also been assumed to be related to longer shopping durations (Niemeier & Morita,^1996^; Schwanen, 2004).

Other influences on the patch residence time and maximum net energy gain rate, such as hunger and nutritional state, fat storage, size, quality and density of food (Busch & Olofsson, 2012; Niemeier & Morita, 1996; Pacheco-Cobos et al., 2019; Schoener, 1971), were difficult to estimate via a questionnaire and were thus not examined due to the setting of this study.

### Questions and Hypotheses

Since the goal of this study is to assess whether supermarkets can be regarded as patches in the sense of the marginal value theorem, we test specific predictions from the simplified MVT model discussed above. If this model is valid, two functional relationships are predicted:

The gain *g(T)* can be described as a function of the patch residence time *T* with the equation $\:g\left(T\right)\:=\:A\:\bullet\:\:(1\:-\:{e}^{-k\:\bullet\:\:T})$, and the function $\:g\left(T\right)$ predicts the travel time *t* with the equation $\:t\:=\:T\:-\:\frac{g\left(T\right)}{{g}^{{\prime\:}}\left(T\right)}$.

Both predictions are tested empirically via regression models and statistical tests. Thus, the exponential growth toward an upper asymptote is fitted as a nonlinear regression model. The predicted and empirical times are subsequently compared via a paired two-tailed t test.

## Method and Procedure

The study was conducted in June 2022 in Braunschweig, Germany. The TU Braunschweig Faculty 2 Research Ethics Committee confirmed that this study required no ethical approval. Braunschweig is a city with an estimated number of 250,495 residents (Feuerwehr Braunschweig, 2020). The supermarket, where the study was conducted, is located on one of the main streets of the city with nearby public transport connections. The participants were randomly approached by a researcher (female, age 22) in front of the supermarket. After verbally providing informed consent, they were questioned about their shopping trip. In particular, they were asked whether they usually shopped at that supermarket. Measuring customers’ travel times and patch residence in different patches of a single supermarket would require an exact means of tracking, which was not possible here due to technical and legal restrictions. Although logging the entry and exit times of participants would have easily provided exact patch residence times, it would not have been feasible to log every person entering the supermarket because not everyone who was approached agreed to take part in the study. Additionally, because both travel time and patch residence time are operationalizations of the same unit (time) and are set (/calculated) against each other, they should be measured in the same way to avoid skewing the calculations. Even if the patch residence times were measured by logging the entry and exit of each participant, the travel times could still have only been estimated due to on-site recruiting. Since it was not possible to physically measure both values, they were both estimated to keep estimation bias as constant as possible. Thus, the main measures of interest were the estimated gains in money spent in *Euro* and costs in *time*.

### Sample

Four people from the original sample were excluded from further analysis because they did not specify their gain, travel time or patch residence time in the survey. The adjusted sample included *N* = 190 persons (110 male, 77 female, 2 nonbinary, 1 agender) between 18 and 90 years of age (*M* = 35.5 years; *SD* = 15.9 years; *n* = 7 not specified). A total of 35.3% of the participants were students, 34.7% were employed full time, 7.9% were employed part time, 7.4% were retired, 4.2% were school pupils, 2.1% were self-employed, 2.1% were trainees, and 6.3% fell into the category of “other”.

### Data Analysis

Data analysis was conducted in R (different versions, latest was 4.3.2) and R Studio Version 4.2.0 (Posit Team, 2023; R Core Team, 2022). Since the variables used in the regression model were the participants’ gain and time *estimates*, it is advisable to model them as including error. To account for this, an orthogonal least squares regression model that minimized orthogonal instead of vertical residuals was used (Spiess, 2022).

The travel time model predictions were determined by the parameters produced in the gain function, and the travel time residuals were examined to analyze the model fit.

For both hypotheses, the residuals of the regression models were inspected visually to check homoscedasticity (residual plot) and their distribution (Q‒Q plot and residual histogram). Following the suggestions of Piñeiro et al. (2008), the observed values were plotted against the predicted values using the intercept as an indicator of model bias.

## Results

### Data Preparation, Selection of a Subset and Operationalization

Our results, as well as further graphics not included here, can be reproduced with the R code. Outlier values were not removed since they could not be assumed to be due to methodological errors. The objective information on distance traveled was excluded from the dataset because of low item acceptance. Moreover, some participants estimated their shopping frequency as varying and had trouble deciding between three or four and five or six times a week. These varying answers were managed by forming categories of shopping frequency. The number of persons in the household provided for by the shopping was categorized because only a few participants reported household sizes of more than two people. Visual analysis of the age distribution revealed some subpopulations that allowed the groups to be categorized by age.

**Fig. 4:**
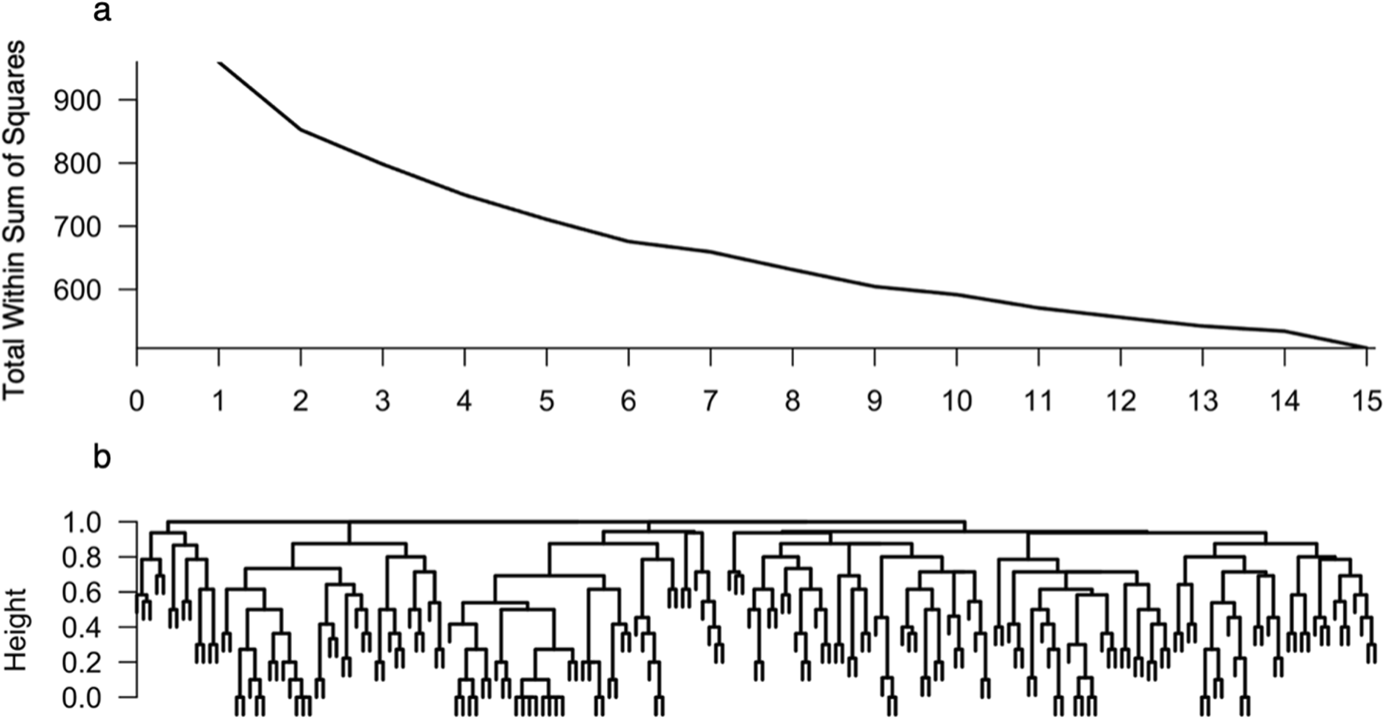
Scree Plot and Explorative Agglomerative Hierarchical Cluster Analysis: Socioeconomic Data. Note: Cluster analysis of dummy variables for the entire dataset (N = 190). The following data from the variables were included: main patch, shopping situation, shopping frequency, number of persons provided for, shopping group size, age, gender, means of transportation, working situation, and exhaustion. Panel **a**: Scree plot for the number of clusters. Panel **b**: Dendrogram.

Before regression models were fitted, explorative agglomerative hierarchical cluster analysis was performed with the aim of identifying homogenous subpopulations, i.e., clusters of similar conditions for which the model could be fitted separately. Cluster analysis of socioeconomic information, which uses the Jaccard distance and a complete linkage method, did not reveal a distinct number of clusters (Fig. 4).

**Fig. 5:**
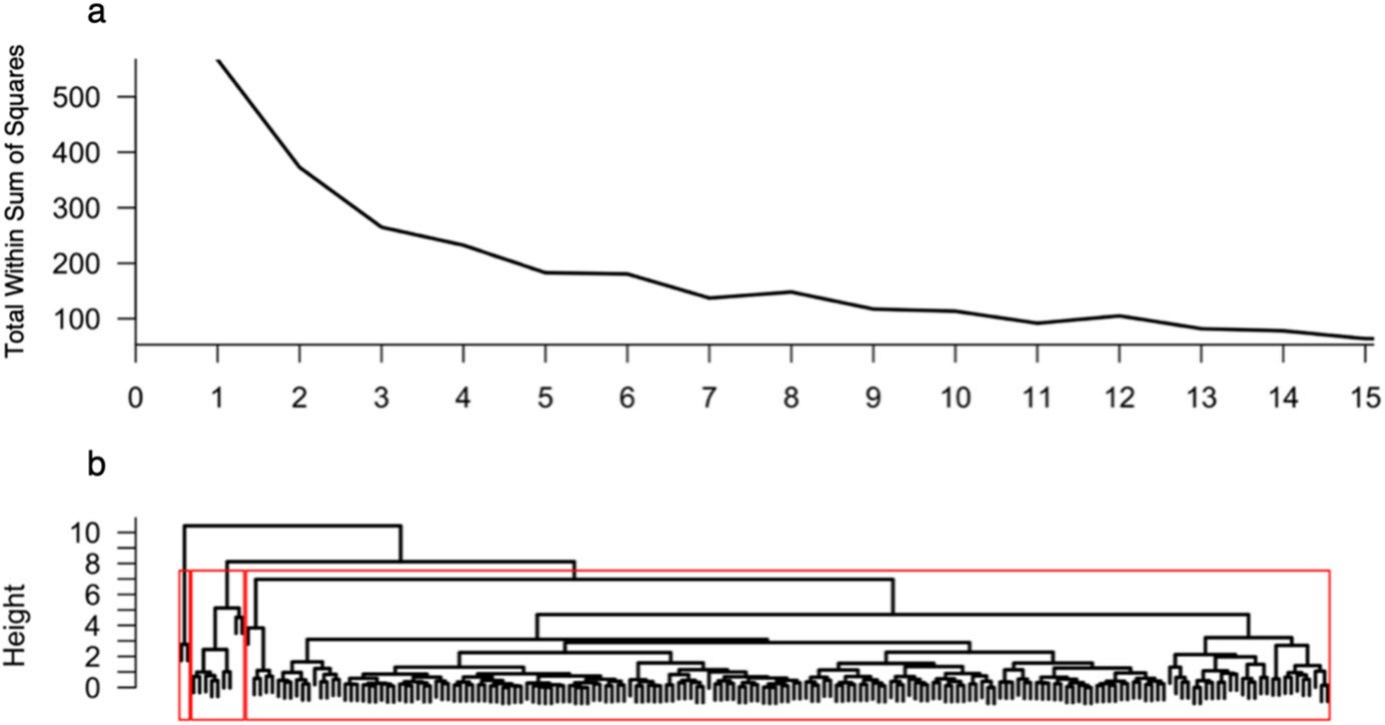
Scree Plot and Explorative Agglomerative Hierarchical Cluster Analysis: Numeric Model Data. Note: Cluster analysis of metric variables for the entire dataset (N = 190). Data from the following variables were included: travel time in minutes, patch residence time in minutes, and gain in Euros. Panel **a**: Scree plot for the number of clusters. Panel **b**: Dendrogram for cluster analysis.

In the cluster analysis of the key variables of the MVT using the Euclidean distance and a complete link method, $\:k\:=\:3\:$ clusters emerged via the elbow method on the basis of the scree plot (see Thorndike, 1953). These clusters, however, did not reflect interpretable variable combinations (Fig. 5).

Therefore, further analysis was not based on clusters within the data structure but focused on a subset of data obtained from theoretical considerations of the conditions for applying the MVT (see *the Marginal Value Theorem* and *Parameters and Measures for the Supermarket Setting section*). The subset consisted of the $\:n\:=\:61\:$ participants who (a) mainly shopped at the supermarket in question and thus had knowledge about the profitability and nature of the patch, (b) traveled from home to the supermarket and back instead of shopping somewhere else on the way and (c) traveled by foot, resulting in a roughly proportional energetic effort for traveling. The conditions of this subset should provide a context in which the model is clearly applicable. The following analyses refer only to this general subset of data.

The following operationalizations of measurement were compared with regard to their model fit, the details of which are beyond the scope of this paper: (a) gain operationalized as money spent in Euros versus the number of products and (b) the travel cost as estimated time versus travel time calculations from distance estimations. The data were analyzed using the operationalizations with the best fit (i.e., the gain in money spent in *Euros* and costs in *time*).

The R code can be used to reproduce the complete analysis for all categories within the subset of *n* = 61 participants who reported shopping mainly at the supermarket in question, traveling from home to the supermarket and back and traveling by foot.

### Gain as a Function of Patch Residence Time

**Fig. 6:**
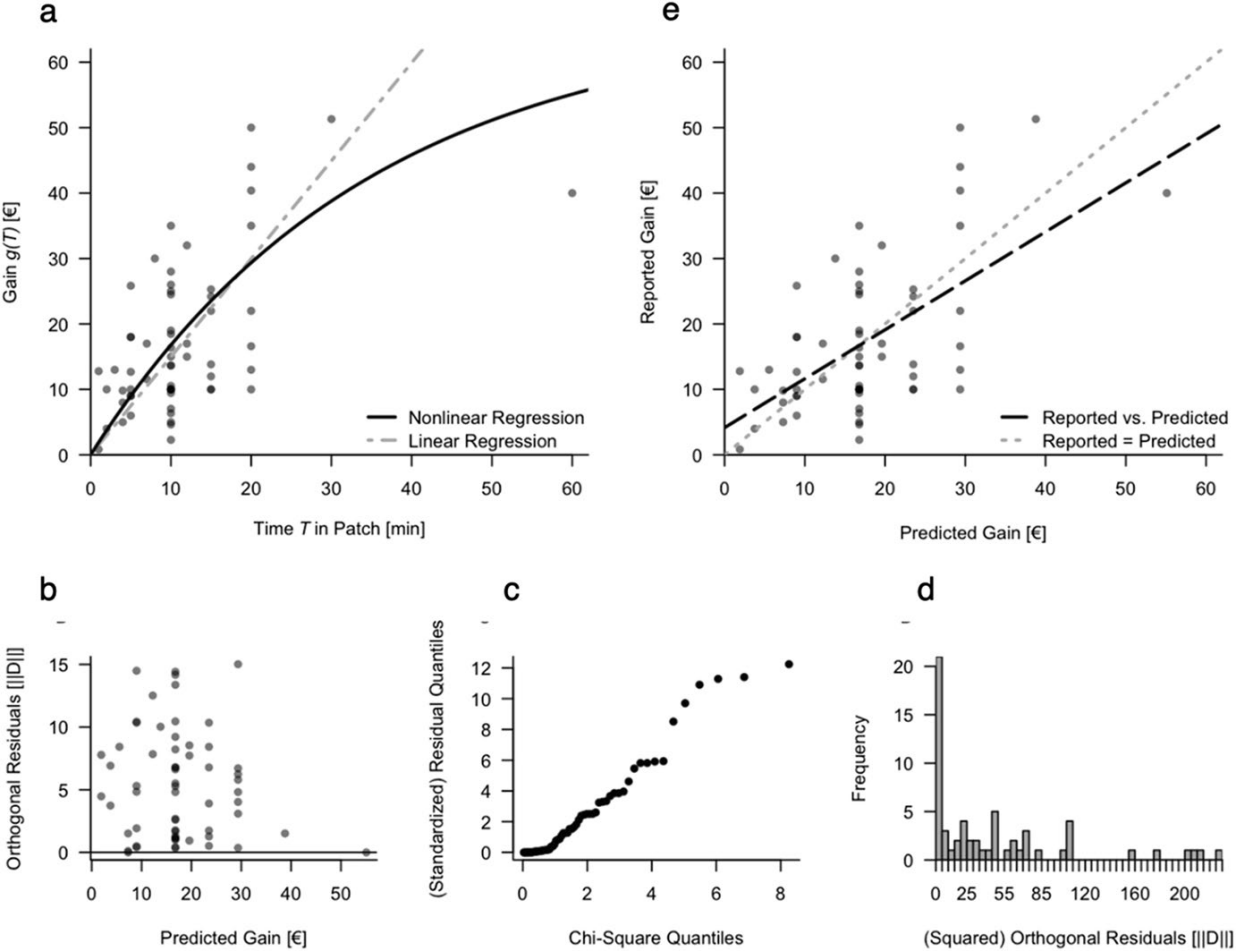
Nonlinear Orthogonal Distance Regression Model for Gain Data (Subset): Data, Residuals, Model Fit. Note: Subset of *n* = 61 participants. Panel a: Nonlinear orthogonal distance regression for gain model $\:g\left(T\right)\:=\:A\:\bullet\:\:(1\:-\:{e}^{-k\:\bullet\:\:T})$ with linear regression added for comparison. Panel b: Predicted gain values plotted against orthogonal residuals. $\:\left|\right|D\left|\right|$: Euclidean length of the orthogonal distance vector. Panel c: Q‒Q plot of squared orthogonal residuals. Panel d: Histogram of squared orthogonal residuals. Panel e: Observed versus predicted gain. The line of perfect prediction has been added in grey. Using the operationalizations selected in the Data Preparation, Selection of a Subset and Operationalization section, an orthogonal distance regression was fitted for gain g(T). For the nonlinear regression in the general subset, SE_residuals_ = 6.99 and s^2^_residuals_ = 48.82

For the general subset, a nonlinear distance regression is depicted in Fig. 6a for the gain as a function of the time spent in the supermarket. Visual inspection of the orthogonal residuals (given by the Euclidean distance from the regression function) of the nonlinear regression model revealed some signs of heteroscedasticity in the general subset with a bias toward greater absolute residuals for smaller predicted gains. The residual scatterplot indicates that smaller predicted gains had larger differences from the observed values (Fig. 6b). Furthermore, the distribution of the residuals was investigated by comparing the squared residuals to a chi-square distribution with two degrees of freedom by means of a Q‒Q plot. This and the histogram of squared residuals suggested a good fit for most of the data, with possible deviations for approximately 4 of the larger observations (Fig. 6c and d). The regression line in the observed-versus-predicted plot shows that for smaller predicted gains, the observed gains were slightly greater than the predicted gains, while for higher predicted gains, the observed gains were slightly smaller than the predicted gains (Fig. 6e). However, an additional analysis with a paired t test revealed no significant difference between the observed and predicted gains (*t* = −0.22, *df* = 60, *p* = 0.838).

### Travel Time as a Function of Patch Residence Time

**Fig. 7:**
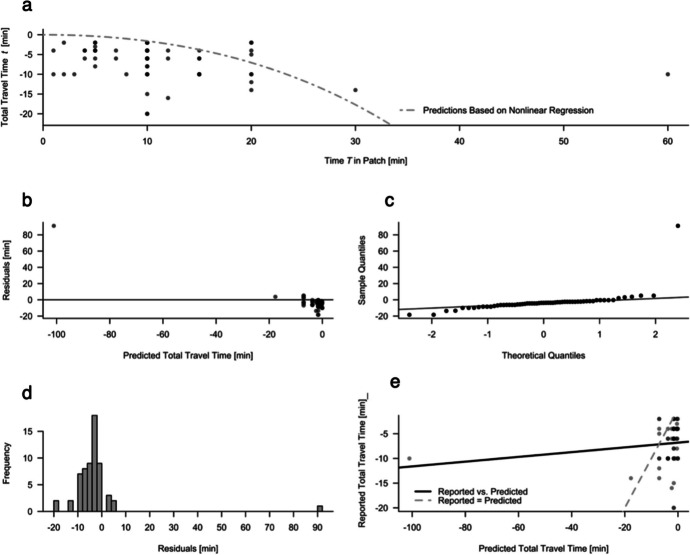
Nonlinear Model Fit for Travel Time Data (Subset). Subset of n = 61 participants. Panel a: Travel times *t*_*j*_ plotted against time *T*_*j*_ spent in the patch. The curve shows model predictions for $\:{t}_{j}\:=\:{T}_{j}\:-\:g\left({T}_{j}\right)\:/\:{g}^{{\prime\:}}\left({T}_{j}\right)$ with parameter estimates from H1a. Panel b: Residuals plotted against predicted travel times. Panel c: Q‒Q plot of residuals. Panel d: Histogram of residuals. Panel e: Observed versus predicted travel times

The predicted and observed travel times for the time spent in the supermarket are modeled with a nonlinear regression for the travel time as a function of the patch residence time, as depicted in Fig. 7a for the general subset. Visual inspection of the residuals for travel times $\:{t}_{j}$ in the nonlinear regression model showed signs of heteroscedasticity with a tendency toward positive residuals for more negative values of the predicted travel times (Fig. 7b). Specifically, this meant longer actual travel times than those predicted by the MVT. The Q‒Q plot and histogram of residuals revealed a fat-tailed, skewed distribution of residuals, indicating that participants tended to travel longer than predicted by their time at the supermarket (Fig. 7c and d). The regression line in the observed-versus-predicted plot showed a tendency toward higher absolute values for reported travel times than for predicted travel times (Fig. 7e). Nonetheless, a paired t test revealed no significant difference between the observed and predicted travel times (*t* = 1.21, *df* = 60, *p* = 0.23). For travel time predictions based on the nonlinear gain regression in the general subset, *SE*_*residuals*_ = 13.17 and *s*^*2*^_*residuals*_ = 173.42.

### Explorative Results

We also investigated group differences in patch residence times via Welch t tests to compare expectations in the case of nonhomogenous population variances. We could compare male and female participants because only one person identified as nonbinary. Furthermore, we compared age groups via a median split method due to categories of insufficient sample size and some missing data. We found no significant differences in patch residence times for different genders (*t* = −1.62, *df* = 25.00, *p* = 0.12) or different age groups (*t* = 0.22, *df* = 54.92, *p* = 0.83).

## Discussion

Despite some theoretical differences between supermarkets and the traditional definition of an MVT patch, our results show that supermarkets are in accordance with the expectations of an MVT model. This is valid at least for participants who (a) mainly shopped at that supermarket, (b) traveled from home to the supermarket and back, and (c) traveled by foot. Therefore, it appears to be possible to apply behavioral ecology models even in urbanized societies.

Although supermarkets are artificially constructed and resource-dense patches that involve smaller levels of analysis for different types of products and corresponding gain units and lack some classical foraging characteristics such as cryptic and stochastic foraging, our simplified MVT model successfully predicts supermarket gains and corresponding travel times.

Although the currency we chose seems appropriate for the application of the MVT, there may be additional currencies for the patch residence time. Individuals may not only maximize the classical measures of the benefit of a resource, such as the energy gain rate, but also other, perhaps social, currencies to maximize their fitness (Nonacs, 2001; Wells, 2012). Another currency that may be maximized while foraging is risk minimization (Morin, 2012). This has been connected to the patch residence time in the MVT context but seems to play a minor role, if any, in supermarkets in Germany at the present time (Nonacs, 2001; Patenaude-Monette et al., 2014). However, perceived risk increases costs in the patch in the context of panic buying and infection risk during the COVID-19 pandemic (Dickins & Schalz, 2020).

Whether organisms optimize or satisfice and settle for approximate local rather than global gains (Simon, 1956) may be a more relevant question for our setting. While our findings are in line with the optimal foraging approach, satisficing behavior generally results in a shorter patch residence time than predicted. This may be connected to findings that suggest that the marginal value is changeable due to intraindividual changes, such as the psychological reinforcement value of items (Laibson, 2001; Waynforth, 2010) or other sociodemographic characteristics. New et al. (2007) reported that the location of resources is remembered better for resources of higher nutritional quality. This may lead to swifter foraging for some resources in supermarkets than for others, resulting in varying patch residence times depending on overall nutritional value. The perceived and actual benefits of a resource may vary inter- and intraindividually and lead to different foraging behaviors (Cassini, 2013). Our approach of assuming monetary value as a measure of resource benefit is simplistic but shows that despite these numerous currencies, simplified approaches can be used to model foraging behavior in supermarkets.

Changes in perceived net gain while in the supermarket (e.g., noticing products on sale that become more appealing than others) and the resulting behavior should be easily predictable by classic prey choice models (e.g., Elner & Hughes, 1978). One would expect foragers to favor prey that gives the highest rate of net energy return. However, this approach cannot be applied when focusing on an entire supermarket as a single patch. Some resources can be quantified in the same gain unit, which allows for direct comparison and prey choice depending on net gain changes due to the available products. However, many products are not comparable because they have different gain units (for example, toilet paper and meat). This is due to the elasticity of demand and the substitutability of resources, which are often studied in behavioral economics (e.g. Rachlin et al., 1976). This can be addressed in the future by examining foraging behavior at the level of smaller patches in supermarkets with different internal structures, which may be defined by products with low substitutability.

The spatial organization of supermarkets may constitute an important contextual factor. New et al. (2007) reported that women performed better at remembering food locations at a farmer’s market regardless of their experience in the patch. Better memory of resource locations within the patch might lead to more efficient foraging and shorter patch residence times. However, in the supermarket setting, we found no significant differences in patch residence times for different genders or different age groups. These groups exhibited very similar foraging behavior.

One distinctive characteristic of many foragers, such as humans, is central place foraging, which increases travel time because we usually do not consume resources in the patch but rather at home and therefore must transport them. In this setting, Houston (2011) highlighted that humans are constrained in their foraging abilities by numerous factors, including their carrying capacity and available energy as well as the duration of time they can be away from their home and temporal constraints within the foraging area. While these are interesting aspects and should be accounted for in the development of more detailed models tailored to the social context in which we live and forage, foraging behavior in the supermarket can be modeled successfully even without accounting for other numeric measures of costs (such as exhaustion) other than time.

In this study, we have made the simplified assumption of an entire supermarket as a single patch. This assumption may be incomplete because different resources might represent different smaller patches within the supermarket. Due to technical and legal restrictions, we were unable to analyze either this internal structure of smaller patches in the supermarket or the behavior within them in more detail. To do so, it would be necessary to observe and explicitly track the behavior of customers (e.g., with predefined coding schemes and measuring instruments). If these restrictions could be addressed, identifying concrete patches and their boundaries, which are likely to vary interindividually, could be an interesting project.

While we found slight though not significant differences in predicted and observed gains and travel times in this study, other studies have reported systematic deviations from MVT predictions in terms of predicted and observed gains and travel times (Cassini et al., 1990; Constantino & Daw, 2015; Cowie, 1977; Nonacs, 2001; Todd & Kacelnik, 1993; Turrin et al., 2017; Venkataraman et al., 2017). The frequency of these findings might indicate that MVT itself may not be adequate, for example, if it involves too few parameters (Nonacs, 2001). However, the question of currencies might also be raised here in relation to the discussion of the marginal fitness gain models above. Additionally, estimates of the model asymptote may depend heavily on single values (cf. Faranda et al., 2020) for an analysis in a different context).

For methodological reasons, the following options are possible:

1. *The actual time spent traveling and in the patch might differ from those reported and assumed here.* Due to the nature of this study, travel and patch residence times had to be estimated by the participants. As a result, the model predictions were not calculated on the basis of fixed values since the predictor variable included errors.
2. *The actual gain function might be different than that assumed here.* Little data were available on larger purchases and large patch residence times or travel times. The shopping trips were mostly short and involved small purchases, and the reported total travel time was never longer than 20 min in the subset of data. Thus, our study lacked data on greater gains and patch residence times, which limits the linear approximation of the nonlinear function. Since this is the interesting part of the curve for more detailed quantitative investigations, the collection of additional data, despite the much higher cost of collection, would be highly valuable.

Despite the established differences between supermarket shopping, common MVT situations and our methodological restrictions, our results demonstrate that supermarket gains and corresponding travel times can be predicted successfully via the MVT. Moreover, the findings imply that these differences are not relevant to human foraging behavior to the extent that supermarket foraging behavior cannot be approximated even by a simplified model based on verbal reports. Therefore, the patch definition of the model can be applied to supermarkets.

The corpus of empirical studies in this area is small, and many contexts remain in which it may be possible to explain human behavior by employing models from behavioral ecology. Various opportunities for pilot studies include not only optimality models but also studies that model human cooperation and other behaviors. For example, Lange and Eggert (2015) conducted a successful field experiment on human cooperation and reciprocity in the supermarket setting.

In our opinion, the next interesting question is whether human shopping, transport and travel behaviors diverge from optimal foraging definitions rather than incompatibility of the supermarket with the patch assumptions of the MVT. While our analysis was based on participants who walked the distance from their home to the supermarket, future studies could investigate changes based on other modes of transportation. This would eliminate the assumption that physical exertion is directly proportional to the energetic and temporal costs of walking and would potentially generating a larger area of application for the MVT.

Subsequent questions could concern additional trade-offs of immediate versus long-term costs (e.g., walking versus car maintenance costs), different supermarket patch densities (i.e., different degrees of urbanization) and severe reductions in either travel costs or costs in the patch (e.g., shopping at two supermarkets next to each other or “clicking and collecting”) in MVT situations.

A promising idea is to use the MVT to predict when to stop consuming a resource as a function of its availability instead of simply the patch residence time. An interesting application of this was Waynforth’s (2010) approach to explain overeating in the context of diets (i.e., regulated food availability). Other directions within foraging theory could be related to the diet breadth model, which employs the same fundamental economic predictions as the MVT and could therefore be used to predict the addition of different types of prey to a forager’s diet (MacArthur & Pianka, 1966; Winterhalder, 1986).

Similarly, one could attempt to model drug abuse and overdose behavior due to the amount of legal regulation by linking optimal foraging to a highly relevant health issue and comparing the predictions to approaches from behavioral economics (e.g. Carroll et al., 1989; Nader & Woolverton, 1992).

Social foraging in groups implies more complex foraging predictions using the MVT. On the one hand, the MVT can predict enhanced security within the group, which leads to lower costs, and division of labor, which leads to higher gains as well as lower costs. On the other hand, social interaction may be a form of currency that potentially leads to a smaller numerical gain than predicted by the model. However, Pacheco-Cobos et al. (2019) demonstrated the successful application of the MVT to group foraging in nature. This may suggest applications in light of the division of labor and separate acquisition of resources by different household members. Attempting to incorporate different investment strategies of partners into a social version of the MVT would also be an exciting next step. Demps and Winterhalder (2019) employed the MVT to weigh the endogenous production costs of resources against the maximum rate of return achieved by exchange with others. These authors further suggested that the inner workings of the rise of barter and trade over the course of human history might be understood within an optimality framework.

Methodologically, this study employed exponential growth toward an upper asymptote as a gain function for the MVT. However, other shapes, such as sigmoidal functions, have been shown to be possible curves that fit the theoretical criteria of the MVT (Stephens & Krebs, 1986; Venkataraman et al., 2017). We further discussed how such considerations can be subsumed in a more general theoretical framework by constructing the function in question as a composition of interpretable feedback and marginal fitness functions. Thus, we linked the MVT model to a new theoretical framework and reviewed its assumptions for an application area and concluded that it may be more broadly applicable than previously assumed. This result is due to the choice of the functions based on limitations not only of the context but also of the foragers themselves (e.g., carrying capacity) and modeling the decaying reinforcer value on the basis of resources that have already been foraged. In this way, we are working toward a better understanding of restrictions that need to be considered when modeling behavior in complex everyday life settings. Addressing theoretical restrictions in pioneering studies is a necessary part of the scientific process and allows for the study of human foraging behavior in larger theoretical frameworks, such as the multilevel model of behavioral selection (Borgstede & Eggert, 2021).

## Data Availability

The dataset analyzed in the current study is available in the OSF repository: https://osf.io/v4xjw/?view_only=966c5c07561f4628be63d266576d9b33.

## Code Availability

The R code is available in the OSF repository: https://osf.io/v4xjw/?view_only=966c5c07561f4628be63d266576d9b33.
